# ADHD: Reviewing the Causes and Evaluating Solutions

**DOI:** 10.3390/jpm11030166

**Published:** 2021-03-01

**Authors:** Luis Núñez-Jaramillo, Andrea Herrera-Solís, Wendy Verónica Herrera-Morales

**Affiliations:** 1 División de Ciencias de la Salud, Universidad de Quintana Roo, Chetumal 77039, Quintana Roo, Mexico; lnunez@uqroo.edu.mx; 2 Laboratorio Efectos Terapéuticos de los Canabinoides, Subdirección de Investigación Biomédica, Hospital General Dr. Manuel Gea González, Calz. de Tlalpan 4800, Belisario Domínguez Secc 16, Tlalpan 14080, Ciudad de México, Mexico; draandreahs@gmail.com

**Keywords:** ADHD, heterogeneous etiology, pharmacological treatment, neurofeedback, qEEG informed neurofeedback, treatment personalization

## Abstract

Attention deficit hyperactivity disorder (ADHD) is a neurodevelopmental disorder in which patients present inattention, hyperactivity, and impulsivity. The etiology of this condition is diverse, including environmental factors and the presence of variants of some genes. However, a great diversity exists among patients regarding the presence of these ADHD-associated factors. Moreover, there are variations in the reported neurophysiological correlates of ADHD. ADHD is often treated pharmacologically, producing an improvement in symptomatology, albeit there are patients who are refractory to the main pharmacological treatments or present side effects to these drugs, highlighting the importance of developing other therapeutic options. Different non-pharmacological treatments are in this review addressed, finding diverse results regarding efficacy. Altogether, ADHD is associated with different etiologies, all of them producing changes in brain development, leading to the characteristic symptomatology of this condition. Given the heterogeneous etiology of ADHD, discussion is presented about the convenience of personalizing ADHD treatment, whether pharmacological or non-pharmacological, to reach an optimum effect in the majority of patients. Approaches to personalizing both pharmacological therapy and neurofeedback are presented.

## 1. Introduction

Attention deficit hyperactivity disorder (ADHD) is a neurodevelopmental disorder (NDD) presenting with inattention, hyperactivity, and impulsivity. It can be classified in three subtypes, depending on the intensity of the symptoms: predominantly inattentive, predominantly hyperactive–impulsive, and combined [1,2]. ADHD has a global prevalence of 5.9% to 7.1% in children and 1.2% to 7.3% in adults [3].

While most studies address ADHD in children from 7 to 17 years old, it is important to outline that this condition is also present in adults. It has been proposed that the number of adults with ADHD has increased over the last 20 years. A part of this increase is due to the permanence of ADHD symptoms in the adult age in 76% of diagnosed patients. ADHD implies important challenges for academic, personal, and job performance [4].

As for any other condition affecting brain function, in order to find an adequate treatment for ADHD, it is important to first understand its physiological basis. As with other NDDs, the causes of ADHD are aberrant neural development, affecting neurogenesis, synaptogenesis, myelination, and neuronal and glial proliferation and migration. Even though symptoms begin to appear in childhood, neuronal development is affected from early embryogenesis [5].

The etiology of ADHD is diverse—gestational, perinatal, and genetic factors have been associated with ADHD incidence. However, each patient presents only a few of them.

## 2. Environmental Factors Associated with ADHD

The incidence of ADHD is associated with a number of environmental factors during different stages of central nervous system (CNS) development, such as gestational and perinatal periods. In this section, we will address some of the environmental factors that have been associated with ADHD.

### 2.1. Preconceptional, Gestational, and Perinatal Conditions

Premature birth is an important risk factor for ADHD, since it has been reported that it occurs 2.6 to 4 times more frequently in babies born with low weight or very low weight. Premature birth is associated with alterations in neurogenesis and cell death [6], and these are in turn associated with reduced cortical expansion, as reported in ADHD patients [7]. One possible reason for increased risk of developing ADHD in preterm children is inflammation; an increase in inflammation-related molecules is associated with increased risk of developing ADHD symptoms [8].

Perinatal hypoxia is an environmental factor that increases the risk of developing AHDH, probably due to its effects on dopaminergic transmission and neurotropic signaling [9].

The intake of nutrients during gestation is very important for proper brain development. An important element during neural development is the polyunsaturated fatty acid docosahexaenoic acid (DHA), promoting proliferation and neural differentiation of neural progenitor cells. Decreased levels of DHA during brain development have been associated with ADHD and other neurodevelopmental disorders [10], and decreased levels of serum DHA levels have been reported in adult ADHD patients [11]. Additionally, malnutrition or immune activation in the pregnant mother is a risk factor for ADHD and other neurodevelopmental disorders [12]. High sucrose consumption during pregnancy is possibly related with ADHD incidence. A study performed on rats reported that high sucrose intake in pregnant rats led to the appearance of ADHD-like symptoms in the offspring, who showed increased locomotor activity, decreased attention, and increased impulsivity. Furthermore, the offspring also presented increased dopamine transporter (DAT) and a decrease in dopamine receptors and mRNA expression in the striatum [13].

Interestingly, there is evidence in a rat model of the influence of preconceptional conditions on ADHD incidence. Offspring of Female rats administered with ethanol for 8 weeks before mating presented ADHD-like symptoms such as hyperlocomotive activity, impulsivity, and attention deficit. These rats also presented low levels of striatal DAT and increased presence of norepinephrine transporter (NET) in the frontal cortex [14]. A later work by this group revealed that paternal preconceptional alcohol exposure also produced ADHD-like symptoms in the offspring, presenting decreased expression of DAT mRNA and DAT protein in the cortex and striatum. Furthermore, authors report epigenetic changes in both the sperm of these alcohol-exposed male rats and in the frontal cortex and striatum of the offspring, presenting increased methylation in a CpG region of DAT gene promoter, which is in agreement with the reduced expression of DAT in the offspring [15].

Another environmental factor associated with ADHD is pesticide exposure during development. A study addressing the issue, both at experimental and epidemiological levels, reported that exposure to the pesticide deltamethrin during gestation and lactation in rats led to ADHD-like symptoms, such as working memory and attention deficits, hyperactivity, and impulsive-like behavior. It also produced increased presence of DAT and D1 receptor in the striatum, as well as increased dopamine release and increased presence of D1 dopamine receptor in the nucleus accumbens. Interestingly, the authors also performed an epidemiological study in humans, revealing that children (6 to 15 years old) with detectable levels of pyrethroid metabolites in urine had more than twice the probability of being diagnosed with ADHD [16].

### 2.2. Heavy Metal Exposure

One of the most reported environmental factors associated with ADHD is exposure to neurotoxic heavy metals. A study performed on school children revealed that children (6–7 years old) with ADHD presented higher levels of salivary mercury. However, when including all age groups studied (12–13 years and 15–16 years), no significant correlation was found between increased salivary mercury and ADHD, although a mild tendency was observed [17].

In the case of manganese, both too high and too low blood levels are associated with cognitive deficits. High concentration of manganese in blood is associated with deficits in thinking, reading, and calculations, as well as with lower learning quotient (indicative of learning disability) and more errors in the continuous performance test (measuring attention and response inhibition). Conversely, low blood level of manganese is associated with a poorer performance in the Stroop test, which is used to assess cognitive inhibition [18]. Similarly, a study addressing the relationship between manganese in drinking water and ADHD found a higher risk of developing this condition (inattentive but not combined subtype) as exposure to manganese in drinking water increased [19]. However, a study on manganese in children’s deciduous teeth failed to find an association between this metal and cognitive deficits [20].

The presence of lead in children’s deciduous teeth is positively associated with hyperactivity or impulsivity, as well as inattention and oppositional or defiant disorder [20]. A study on children from a lead-contaminated region reported that blood levels of cadmium, lead, and manganese correlated with conduct problems and antisocial behavior [21]. Another work found a higher concentration of blood lead in ADHD children, which was correlated with hyperactivity–impulsivity symptoms but not with inattention [22,23]. Both genetic [24] and epigenetic [25] factors have been reported to contribute to lead-related pathogenesis of ADHD. Moreover, a study carried out in Argentina found that children with high blood concentrations of lead are more likely to develop ADHD [26].

A review on the effects of prenatal and childhood metal exposure on cognition found suggestive evidence of a relation between cadmium exposure and impaired cognitive ability in children. They did not find evidence of a relationship between cadmium exposure and ADHD [27]. A more recent study addressing cadmium exposure during pregnancy revealed that a higher blood cadmium concentration during pregnancy is associated with higher scores in ADHD diagnostic tests in female children at 6 years of age, but not in the case of male children [28].

A recently published work reported that ADHD children present higher urine concentrations of chromium, manganese, cobalt, nickel, copper, molybdenum, tin, barium, and lead [29]. A recent study analyzing serum concentrations of different metals in ADHD children reported low levels of chromium, manganese, and zinc, as well as increased copper/zinc ratios in these children [30]. A meta-analysis on the relation between blood and hair zinc and ADHD found no statistical difference between ADHD and control children [31].

Thus, there are a number of environmental factors associated with ADHD incidence. While environmental factors are not found in all ADHD cases, the data reviewed herein highlight the importance of environment in different developmental stages—and even before conception—in regard to the risk of developing ADHD.

## 3. Sleep Disorders and ADHD

Sleep deprivation, either acute or chronic, produces decreased cognitive functioning (one of the main traits of ADHD). Interestingly, it also produces the externalizing symptoms observed in ADHD patients. For example, a very tired child might become hyperactive, while in a sleepy adult in a condition where it is not possible to sleep (for example, while driving), the externalizing behavior will help them to remain awake. Thus, both of the core ADHD symptoms can be produced by sleep deprivation. Conversely, hyperactivity in children or high internal activity in adults in the evening might lead to sleep disruption [32].

Among the sleep disorders found in ADHD patients are delayed sleep phase disorders, insomnia, sleep-disordered breathing, increased motor activity during the night, sleep anxiety, clenching teeth, periodic limb movement, restless legs, increased sleep onset latency and shorter sleep time, night awakenings, narcolepsy, and parasomnias [32,33,34,35,36,37,38,39]. Among them, delayed sleep phase disorder is one of the most frequently found, being present in 73–78% of both ADHD children and adults. This condition consists of a delay between the sleep propensity cycle and the circadian cycle, leading to increased daytime sleepiness and decreased cognitive functioning [32].

Sleep disturbance have an impact on daytime vigilance, producing excessive sleepiness [32,37,39], and can exacerbate inattention, impulsivity, and hyperactivity as means to remain awake [32,37]. Additionally, stimulant medication might also cause sleep disturbances, although OROS methylphenidate produces less adverse effects on sleep [34,36]. LDX, a stimulant prodrug that undergoes hydrolysis in the bloodstream releasing d-amphetamine, and atomoxetine, a non-stimulant pharmacological treatment for ADHD, do not produce adverse effects on sleep [36]. 

Sleep disturbances in ADHD patients can produce significant impairments in attention, mood, and behavior [32,35]. Physiologically, there is evidence supporting an overlap between brain centers regulating sleep and those regulating attention and arousal, so it is possible that affectation of one of these systems also affects the other. Similarly, affectation of noradrenergic and dopaminergic pathways is found in both ADHD and sleep disturbances [40].

Conversely, during wake time, sleep disturbances produces symptoms resembling those observed in ADHD patients [35,41,42]. It is, thus, recommended to assess sleep disorders in patients with ADHD symptoms in order to avoid misdiagnosis [41,42].

The relationship between sleep disorders and ADHD is complex. While ADHD might produce sleep disorders, they could also be coincident conditions [36]. Moreover, sleep disorders have been proposed to be not only one of the intrinsic features of ADHD, but also might be one of its causes [32,36]. Another possible explanation for this interaction would be an underlying common neurological disease leading to both sleep disorders ad ADHD [36]. A recent review on the subject proposed that chronic sleep disorders are some of the main causes of ADHD symptoms [32]. The authors suggested that patients presenting ADHD symptoms should undergo quantification of sleep and sleep problems in order to rule them out as the sole cause of ADHD symptoms. Thus, ADHD treatment should address both the symptoms (with classic ADHD treatment) and the sleep problem [32,34,35,36], although the effect of this combined treatment still requires further research [32].

## 4. Genetic Factors Associated with ADHD

Different studies have revealed an important genetic influence in the etiology of ADHD [43]. It is a polygenic condition with an important number of genes involved, as confirmed by a genome-wide association study on ADHD reporting 12 significant loci associated with this condition [44]. Many of the genes reported to be associated with ADHD participate in processes such as neurotransmission, neuritogenesis, synaptogenesis, or receptor location in synapses [45]. In this review, we will focus on two genes, a neurotrophin (brain-derived neurotrophic factor –BDNF-) and a molecule involved in dopaminergic signaling (DAT).

### 4.1. BDNF

Brain-derived neurotrophic factor (BDNF) is a neurotrophin with high expression in the brain that is highly concentrated in the hippocampus and cortex. It has an important role in neuronal development, being important for neuronal proliferation, migration, differentiation, and maturation, as well as for synaptogenesis [46]. 

BDNF has been implied in ADHD pathophysiology. It has been proposed that low levels of this neurotrophin may explain the reduction in brain volume observed in ADHD patients, and it has also been implied in dopaminergic system homeostasis. Some pharmacological treatments for ADHD promote the regulation of plasma BDNF levels [47]. 

#### 4.1.1. Circulating BDNF 

Since BDNF is able to cross the blood–brain barrier and plasma concentrations of BDNF are highly correlated with its levels on cerebrospinal fluid, a number of studies have searched for a difference in plasma concentrations of BDNF in ADHD patients when compared against controls. There are reports indicating a lower concentration of BDNF in plasma of ADHD patients, both in children [48] and adults [49]. In another study involving children, an increase in plasma BDNF was observed after 6 weeks of treatment with an effective dose of methylphenidate [50]. In accordance, a recent study revealed that methylphenidate treatment produces an increase in serum BDNF in boys with ADHD [51]. However, this has not always been replicated, since there are also articles reporting no difference in serum BDNF between children with ADHD and controls [52,53,54].

A recently published meta-analysis encompassing studies comparing BDNF levels in ADHD patients without any other comorbidity found no overall difference between ADHD patients and controls. However, when analyzing males and females separately, they found significantly higher levels of plasma BDNF in males with AHDH than in control males, while no difference was found between females with and without ADHD [55]. 

Thus, different and even contrary results have been obtained regarding BDNF concentrations in plasma or sera of ADHD patients. While this suggests that the link between BDNF and ADHD is not completely clear, other alternatives should be considered. For example, fluctuations in serum BDNF concentrations in morning and evening samples have been reported [56], meaning the lack of relation between peripheral BDNF concentration and ADHD might be due to the time of the day when the sample was obtained. 

#### 4.1.2. Genetics of BDNF

There are a number single nucleotide polymorphisms (SNP) of the BDNF gene that have been associated with ADHD. Among the most studied variations in the BDNF gene, there is a polymorphism called Val66Met (also known as rs6265), in which a change in codon 66 produces a substitution of the original amino acid (valine) by methionine. The anatomical effects of this variation are more apparent in the hippocampus and cortex [46]. While some studies have assessed the presence of this SNP in ADHD patients [57,58,59], other studies failed to find an association between this polymorphism and ADHD [46,60,61,62,63].

Another SNP of the BDNF gene whose association with ADHD is not conclusive is rs2030324, since some studies report an association between this polymorphism and ADHD [57,58,59,64], while other reports fail to find this association [46,60,61,62,63].

There are other SNPs of the BDNF gene that have been studied so far, with positive correlations being shown between ADHD and the presence of C270T (rs27656701) [58,61], rs11030101 [62,64,65], and rs10835210 [62,63]. There are also reports addressing SNPs of the BDNF gene for which no association with ADHD has been found, including rs12291186, rs7103411 [63], and rs7103873 [62,63].

Moreover, rare single nucleotide variants of BDNF gen have also been associated with a higher risk of developing ADHD [66]. However, this is an area that requires further research.

As observed with peripheral BDNF concentrations, genetic variants of the BDNF gene have been associated with ADHD in numerous cases, although in some cases there are contradictory results in different articles (see Table 1). Moreover, some of the genetic variants of the BDNF gene associated with ADHD have also been studied in association with other neurological conditions and treatments. For example, C270T is reported to be associated with intellectual disabilities [58]. Moreover, rs11030101 is associated with a better response to electroconvulsive shock therapy for treatment-resistant depression [67], with body weight gain in schizophrenic patients treated with atypical antipsychotics [68], as well as with the presence of major depressive disorder [69], schizophrenia, and bipolar disorder [70], although there is another publication in which no evidence of association between this SNP and bipolar disorder was found [71]. Additionally, rs10835210 has been associated with bipolar disorder, schizophrenia [70], and phobic disorders [72].

For rs6265 (Val66Met), there are many articles addressing the association of this SNP with different conditions, and in some of them it has been found. For example, some articles report an association of this SNP with major depressive disorder [69,73], while other studies fail to find this association [74,75]. An association has also been reported between rs6265 and amnestic mild cognitive impairment, as well as with the transition from this condition to Alzheimer’s disease [76]. However, in patients with early-stage breast cancer, this SNP is associated with a lower probability of presenting cognitive impairment after chemotherapy [77].

#### 4.1.3. Other Neurotrophines

While BDNF has been widely studied in association with ADHD, it is not the only neurotrophin studied in relation with this condition, given the important role of neurotrophines in central nervous system development and synaptic plasticity. In this regard, there are studies addressing the participation of fibroblast growth factor (FGF), vascular endothelial growth factor, insulin-like growth factor (IGF2) [47], glial-derived neurotrophic factor (GDNF), nerve growth factor (NGF), and neurotrophin-3 (NTF-3) [47,53] in ADHD pathophysiology.

BDNF is a molecule highly involved in synaptic plasticity and has an undisputed role in central nervous system development. Therefore, it is not surprising to find a number of studies associating alterations in the presence of this neurotrophin in serum, or different SNPs of its gene, with ADHD. However, its role in ADHD development is not a constant for every sample of ADHD patients studied so far, and for many of the aspects of this molecule (serum levels, SNPs) there are reports indicating associations, with others finding no association at all. This does not mean that the alterations associated with this molecule are not important for ADHD, but rather highlight the variable etiology of this condition. 

### 4.2. Dopaminergic System 

The dopaminergic system emerges in early stages of CNS embryonic development, and an imbalance in this system might affect brain development. It is related with cell proliferation, neuronal differentiation and migration, synaptogenesis, and neurogenesis. Thus, it is not surprising that a role of this neurotransmitter system has been reported in different neurological diseases, including ADHD [78].

One of the most studied molecules of the dopaminergic system in relation to ADHD is DAT, a molecule responsible for dopamine reuptake, and the main target of two commonly used pharmacological treatments for ADHD, methylphenidate and amphetamines [78]. Genetic studies support the importance of this neurotransmission system for ADHD. Mice heterozygous for the DAT gene (+/− heterozygotes) are reported to present altered attentional function [79,80] and hyperactivity [80], while rat models with this heterozygous genotype do not present major affectations [81,82]. However, DAT knockout rats present hyperactivity [81,82], as well as a dysregulation in frontostriatal BDNF function [82]. Hyperactivity in these rats can be counteracted by amphetamine, haloperidol, and methylphenidate [82].

In humans, ADHD patients present lower DAT availability in the basal ganglia, caudate nucleus, and putamen [83]. The DAT gene presents a variable tandem repeat region (VNTR) at the untranslated 3′region, and there are different alleles for this VNTR, with the 9-repeat and 10-repeat alleles being the most frequently encountered. The reported effects of this VNTR on DAT expression vary in different articles, however the most recent results indicate that the 9-repeat allele is associated with a higher DAT expression than the 10-repeat allele. [84]. The possible association between this VNTR and ADHD has been addressed in various studies. For example, an analysis of both patients and the literature found an association of the 10-repeat/10-repeat genotype with ADHD only in adolescents [85], studies performed in children reported an association between the 10-repeat/10-repeat genotype and ADHD [86,87], while a recently published meta-analysis reported an association of the 10-repeat allele with ADHD in children and adolescents, specifically in European population [88]. However, there are also reports indicating no association at all between ADHD and the VNTR of DAT gene (9-repeat/10-repeat, 10-repeat/10-repeat, and 10-repeat/11-repeat genotypes) [89], no association between the 10-repeat/10-repeat allele with ADHD [90], and no association between ADHD and the 9-repeat or the 10-repeat alleles for this polymorphism [91]. The last three studies were performed in children. 

Additionally, the relevance of this VNTR has been studied in relation to cognitive function in healthy subjects. Again, mixed results were found. A meta-analysis published in 2016 addressing studies performed in healthy subjects did not find any association between DAT VNTR and different cognitive functions, such as executive functions, inhibition, attention, and long-term declarative memory [92]. A study performed in children aged 3 to 5 years old addressing the presence of the 9-repeats and 10-repeats alleles revealed that the presence of the 10-repeat allele of the DAT gene is associated with diminished ability to voluntarily regulate reactivity in healthy children [93]. A recent study on both ADHD and healthy children reported an effect of the specific genotype in the performance of children on attentional switching when studying the whole research sample, in which children carrying the 9-repeat allele performed worse than those carrying the 10-reapet homozygous or the 10-repeat/11-repeat heterozygous allele [91]

The participation of the dopaminergic system in the pathophysiology of ADHD has been widely reported [78]. Herein, we study a particular variation of the DAT gene, a VNTR in the 3′ region of the gene, finding articles supporting a role of this polymorphism in ADHD, as well as works failing to find an association between this VNTR and ADHD. This does not imply a lack of importance of this variation, but rather highlights the variability in the genetic etiology of this condition. Moreover, while the dopaminergic system is highly involved in the pathophysiology of ADHD, given its role in CNS development, it is also strongly related with other neuropsychiatric conditions, such as autism [78,94,95] and schizophrenia [78,94,96,97].

## 5. Changes in Brain Structure and Function in ADHD Patients

As an NDD, ADHD involves alterations of mechanisms such as neurogenesis and synaptogenesis. There are a number of possible mechanisms through which these alterations take place, both environmental and genetic, some of which have been mentioned in the present review. In the end, all of these altered mechanisms produce an altered brain function affecting attention and impulse control, functions regulated by the central nervous system. Understanding the changes in brain function associated with ADHD might shed some light not only on the functional causes of this condition, but also on possible ways to deal with it.

### 5.1. Brain Imaging Studies

Children with ADHD present atypical connectivity in reward circuitry when compared with control children. Increased connectivity of the nucleus accumbens with the prefrontal cortex was observed to be associated with greater impulsivity [98].

Hypofunction and abnormal cortico-striatal pathways of the cortico-striato-thalamo-cortical (CSTC) circuit are associated with ADHD. Five different CSTC circuits have been reported: the sustained attention circuit, emotion circuit, selective attention circuit, hyperactivity circuit, and impulsivity–compulsivity circuit. Four of them (except emotion circuit) have been related with ADHD diagnostic criteria. However, pathogenesis of the emotion circuit is also related with ADHD [99]

A study on ADHD children reported significantly decreased white matter volume, as well as decreased volume in the cortex and caudate nucleus, although it did not reach statistical significance. Cortical thickness was reduced in ADHD patients bilaterally in the frontal cortex and in the right cingulate cortex, structures related with executive function and attention. Regarding default mode network, functional connectivity was reduced in ADHD children in the anterior and posterior cingulate cortexes, lateral prefrontal cortex, left precuneus, and thalamus. However, connectivity was increased in the bilateral posterior medial frontal cortex [100]. 

A study on male adolescents with ADHD and controls reported decreased gray matter volume in the left anterior cingulate cortex and bilateral decreases in the occipital cortex, hippocampus–amygdala complex, and cerebellum in ADHD adolescents [101]. Such decreases in cerebellar volume have been previously reported in both female and male ADHD patients [102].

An important issue with many of the imaging studies in ADHD patients has been small sample size. A large-scale study performed on children, adolescents, and adults with ADHD reported decreased surface area in children, mainly in the frontal, cingulate, and temporal regions. This effect was more pronounced in younger children (4–9 years old). Moreover, cortical thickness in ADHD children is also reduced in the fusiform gyrus and temporal lobe, an effect more prominent in children of 10 and 11 years old. No change in surface area or cortical thickness was observed in adolescent or adult ADHD patients [103].

There are important changes in brain morphology in ADHD patients. An elegant study performed in ADHD patients and controls from 6 to 28 years of age analyzed differences in neurodevelopmental trajectories. This study reported that ADHD patients present overall reduced cortical volume, mainly in frontal lobes, and primarily due to a decrease in surface area and gyrification. Interestingly, although both groups presented maturational changes due to age, they presented different trajectories for these changes, suggesting that ADHD is associated with developmentally persistent changes in the whole cortex, mostly due to decreased surface expansion (reduced surface area and less convolution) [7].

When comparing children with comorbid epilepsy and ADHD with control children, a widespread decrease in cortical thickness is observed, along with decreased volume in some subcortical structures and the brainstem. These alterations were observed early in the course of epilepsy, thus the authors suggested that neurodevelopmental changes occurred before epilepsy onset [104]. In children with comorbid autism spectrum disorder and ADHD, when compared with typically developing controls, presented significantly lower volumes in left postcentral gyrus. This was observed through magnetic resonance imaging in both children and preadolescents, but was absent in adolescents. The authors suggested that pathophysiology in these comorbid patients may be related to somatosensory deficits and delayed maturation in this area [105]. 

### 5.2. Quantitative Electroencephalography

All these changes lead to alterations in brain function. A frequently used technique for the study of brain activity is quantitative electroencephalography (qEEG), since it has a low cost, a high temporal resolution, and does not need special facilities to be performed. Furthermore, qEEG has also been used to determine the effects of pharmacological treatments on brain activity in order to assess effectiveness [106,107], to choose the correct pharmacological option for a patient [108], to study the effects of previous pharmacological treatments on the current one [109,110], as well as to determine a possible cognitive effect of the chosen pharmacological treatment [111].

During the last decades, several studies have performed qEEG analyses on ADHD patients. A review on the subject published in 2012 addressed the main associations between brain activity and ADHD, including increased frontocentral theta activity. Another frequently reported factor, although not always replicated, is an increased theta/beta ratio. For beta and alpha bands, most of the reports have indicated decreased activity, although there are also reports that have indicated increased activity in these frequency bands in ADHD patients [112]. One of the most used indicators for ADHD is the theta/beta ratio in the Cz region. It has been reported that ADHD children (inattentive and combined subtypes) present increased theta/beta ratios [1]. Another study found that children with ADHD presented more delta and theta activity [113]. However, some authors have mentioned that this measure is not necessarily useful for diagnosis, since among other issues, it presents variations according to age [114]. 

Another example of the influence of age on brain electrical activity associated with ADHD is a study comparing children with and without ADHD, as well as adults with and without ADHD. Interestingly, children with ADHD presented higher delta and theta activity than control children, while in adults no difference was found between ADHD group and controls in the frequency bands analyzed [115]. Among the few differences in qEEG activity found in adults with ADHD is a higher gamma activity (39.25–48 Hz), suggesting a functional alteration in dorsal attention network [116].

ADHD patients often present comorbidities [117], which might influence qEEG in a different way to the findings in ADHD only patients. For example, children with ADHD and problematic Internet use present differences in qEEG when compared to ADHD only patients. However, no differences were found between ADHD only patients and ADHD patients with depression [118]. Another study found that adolescents with ADHD and Internet gaming disorder presented lower relative delta power and greater relative beta power than adolescents with ADHD only [119].

It is noteworthy that although a number of studies have been published regarding neurophysiological correlates of ADHD through qEEG, there are still some differences in the results reported by different authors. Beyond possible methodological differences, there are a number of factors reported to influence qEEG activity in ADHD patients, which might be responsible—at least in part—for the differences reported so far, and which might be of importance when using qEEG information to choose or design a therapeutic approach. These factors include comorbidities [4,120] and the ages of the patients [114,116,121]. Other factors reported to affect qEEG activity in other populations and conditions are ethnicity [122,123,124,125,126], sociocultural environment during development [127,128], and the degree of advancement of a psychiatric condition, as reported for alcohol dependence [129,130,131].

## 6. Therapeutic Approaches

### 6.1. Pharmacological Treatment

Both stimulant and non-stimulant pharmacological treatments have proven to be effective in diminishing ADHD symptoms in children and adolescents [132,133], although stimulant medication seems to have greater effectiveness [133,134]. Herein, we will address one frequently used stimulant (methylphenidate) and one frequently used non-stimulant (atomoxetine)

#### 6.1.1. Methylphenidate

Methylphenidate is one of the most used drugs for ADHD treatment. It has been present in the market for 50 years and it reduces excessive hyperactivity, impulsivity, and inattention in children and adolescents with ADHD. In the United States, it is prescribed to 8% of children and adolescents under 15 years of age and to around 3% to 5% of the same population in Europe [135]. 

Methylphenidate blocks DAT and NET, reducing reuptake and producing an increase in available dopamine and norepinephrine in the synaptic cleft [135,136,137], leading to increased dopamine and norepinephrine transmission in the prefrontal cortex [132]. A meta-analysis on the effects of methylphenidate treatment on ADHD in adults found it effective in improving neurocognitive performance, accomplishing better results than placebo groups in terms of working memory, reaction time variability, vigilance, driving, and response inhibition [136].

#### 6.1.2. Atomoxetine

Atomoxetine has been reported to be effective for ADHD treatment [138], being more effective in adults than in children [134]. 

Atomoxetine blocks norepinephrine reuptake, producing increased presence of norepinephrine and dopamine in prefrontal cortex [132]. Since atomoxetine does not produce an increase of dopamine or norepinephrine in the nucleus accumbens, it lacks abuse potential [132,139]. This drug is associated with improvements in quality of life in children adolescents and adults, although this parameter is not further increased with long-term use [139].

#### 6.1.3. Adverse Effects

Both stimulant and non-stimulant pharmacological treatments for ADHD produce adverse effects in a percentage of treated patients. The main adverse effects found for these drugs (% of patients treated with stimulants/% of patients treated with non-stimulants) are decreased appetite (28.6%/14.2%), nausea (7.9%/10.3%), headache (14.5%/20.8%), insomnia (12.3%/8.6%), nasopharyngitis (6.0%/7.1%), dizziness (5.1%/10.0%), abdominal pain (7.8%/11.5%), irritability (9.3%/6.9%), and somnolence (4.4%/34.1%) [133].

A systematic review on the adverse effects of methylphenidate in children and adolescents revealed that about 1 in 100 patients present serious adverse events after methylphenidate treatment (including death, cardiac problems and psychiatric disorders), while more than half of the patients treated with methylphenidate suffer one or more adverse events. The authors concluded that it is important to identify subgroups of patients who might be harmed by methylphenidate treatment and highlight the importance of remaining alert to possible adverse events in patients with this treatment [135]. There might also be uncommon adverse effects. For example, there is a report of 3 cases of systemic sclerosis associated with methylphenidate treatment [140]. The authors of the last study suggested that patients with signs of autoimmune or vasospastic conditions should be briefed about this possible side effect before commencing methylphenidate treatment.

A systematic review on possible adverse effects of atomoxetine, including decreased growth rate, cardiovascular and hepatic effects, aggression, psychosis, seizures, and suicidal ideation, determined that evidence indicates it is safe to use in ADHD patients [141]. Furthermore, the presence of comorbidities does not interfere with treatment efficacy, nor does treatment exacerbate comorbid symptoms [142,143]. However, it is important to be alert to other possible adverse effects. A case report and review indicated that the appearance of tics is a common side effect of atomoxetine treatment [144]. 

Methylphenidate and atomoxetine are known to increase heart rate and blood pressure, raising concern regarding possible cardiovascular effects of these drugs in ADHD patients. A review on the cardiovascular effects of these drugs in healthy subjects found the drug to be safe to use. Most of these studies were performed in children and adolescents, although there have also been some studies performed on adults, with no serious risk being reported in these subjects either. However, patient blood pressure and heart rate should be monitored on a regular basis. Moreover, careful follow-up should be performed for patients presenting certain cardiovascular conditions [145]. 

Weight loss has also been reported after atomoxetine treatment, occurring during the first two years of treatment. However, evidence suggests this decrease begins to be compensated between 2 and 5 years after the beginning of treatment [141]. Similarly, methylphenidate has been associated with adverse effects such as anorexia, weight loss, and insomnia [146].

A comparative study on short-term effects of methylphenidate and atomoxetine on ADHD reported significantly higher weight loss in children treated with atomoxetine [147]. However, a more recent study reported that children present significantly more weight loss after methylphenidate than after atomoxetine treatment [148].

A meta-analysis on gastrointestinal adverse effects of methylphenidate reported increased risk of decreased appetite, weight loss, and abdominal pain in children and adolescents under this pharmacological treatment [149].

A comparison between the presence of adverse effects after methylphenidate and atomoxetine treatments in ADHD children indicated methylphenidate as a safer option, since children under atomoxetine treatment presented higher incidence rates of anorexia, nausea, somnolence, dizziness, and vomiting than children under methylphenidate treatment [147]. A more recent study reported similar results, since children treated with atomoxetine presented higher incidence rates of mild adverse effects, such as decreased appetite, weight loss, dyspepsia, abdominal pain, stomach ache, irritability, mood disorders, and dizziness. As for severe adverse effects, patients under atomoxetine treatment presented higher incidence rates of gastrointestinal, neuropsychiatric, and cardiovascular effects [150].

#### 6.1.4. Long-Term Adverse Effects

Long-term adverse effects of methylphenidate are the subject of intense study, given that it is the first-line stimulant drug used for ADHD treatment in children, adolescents, and adults [11,151]. A review on the subject addressed different adverse effects studied in patients after long-term (over one year) administration of methylphenidate, including low mood or depression, anxiety, irritability or emotional reactivity, suicidal behavior or ideation, bipolar disorder, psychotic symptoms, substance use disorders, tics, seizures or EEG abnormalities, and sleep disorders. The authors concluded that existing information indicates that methylphenidate is safe to use, although caution should be taken when prescribing this drug to specific groups, such as preschool children, patients prone to psychosis or tics, and high-risk adolescents [152]. However, the need for more studies on the long-term effects of treatment with this drug is highlighted, since studies in humans are rather scarce and with a high degree of heterogeneity in terms of methodological approach [151,152].

#### 6.1.5. Long-Term Therapeutic Effect

Given that ADHD is a chronic disorder and that many of the children presenting ADHD will still present symptoms in adulthood, it is particularly important to determine the long-term effectiveness of pharmacological treatments. However, very few studies address this issue, and no conclusion can yet be drawn regarding the long-term effects (years) of pharmacological treatment of ADHD on symptom reduction and quality of life. Thus, the long-term efficacy of drug treatment for ADHD remains under debate [153,154,155,156]

Current pharmacological treatments for ADHD have proven to be safe and effective. The efficacy of these treatments on ADHD symptoms is clear, and thus pharmacological therapy is often used to treat ADHD patients [136,138,141,152]. However, there are also some drawbacks to this therapeutic approach, including the time required to reach the effective dose for each patient [3,157]; the lack of response in some patients [121,158,159,160]; the unresolved issue of long-term effectiveness (of great importance given that in many cases the treatment must go on for years) [153,154,155,156]; the presence of adverse effects, which although not life threatening in most cases, are nevertheless upsetting [133,135,144]; and the existence of specific groups of patients with whom a greater caution must be taken [140,145,152]. Altogether, these drawbacks have led to the search of new therapeutic approaches. One of the strategies studied so far is the possibility of using other drugs to treat ADHD, including drugs interacting with serotoninergic (metadoxine, paroxetine, duloxetine, buspirone), glutamatergic (memantine), cholinergic (AZD3480, AZD1446, lobeline, galantamine, mecamylamine), histaminergic (mk-0249), and catecholaminergic neurotransmission systems (modafinil, droxidopa, desipramine, bupropion, nomifensine, reboxetine, venlafaxine, duloxetine, guanfacine, aripiprazol, dasotraline, selegiline), as well as lithium [161].

### 6.2. Non-Pharmacological Therapies

Pharmacological therapy is effective although presents some inconveniences, including the existence of adverse effects in some patients and lack of effect in others. Therefore, there are also different non-pharmacological approaches for ADHD treatment.

#### 6.2.1. Behavioral Parent Training

The goal of parent training is to equip parents with techniques that will be useful in managing ADHD-related behavior presented by their children. A systematic review published on 2011 found no reliable effect of ADHD children’s behavior, although it may lead to increased confidence and decreased stress in parents [162]. Later studies found an effect of behavioral parent training on ADHD symptoms, which is not increased by previous working memory training, although this combination did produce positive effects on working memory storage and processing [163]. It is noteworthy that cognitive functioning of both parents and children influences the effectiveness of this therapeutic approach on ADHD symptoms. Better working memory in children and higher parental response caution presented an association with improvements in inattention. As for conduct problems, better parental self-regulation was associated with a better result in this area. However, none of the measured cognitive functions in children or parents were associated with improvements in hyperactivity [164]. Moreover, behavioral parent training improves coexistence at home, since a reduction in the frequency and severity of problematic situations is produced, along with a reduction of stress in parents [165].

#### 6.2.2. Cognitive Behavioral Therapy

Cognitive behavioral therapy (CBT) has also been used to treat ADHD. A review performed on the subject found CBT to be effective in reducing ADHD symptoms in adults, however only when improvement was evaluated by the patient and not when evaluated by the clinician [166], although a more recent meta-analysis on the subject reported a good effect of CBT on ADHD adults [167]. A Cochrane systematic review concluded that CBT has a positive effect on ADHD symptoms, either alone or in conjunction with other therapies, although considered the evidence to be low-quality in accordance with the Grading of Recommendations Assessment, Development and Evaluation (GRADE) working group approach. [168]. A meta-analysis found that CBT is one of the most effective non-pharmacological options to treat ADHD, ranking just after physical exercise [169]. A later systematic review confirmed the effects of CBT on ADHD symptoms [170]. A recent study reported CBT to be effective in reducing ADHD symptoms in patients, either with or without conjunct medication [171].

#### 6.2.3. Attention Training Techniques

Attention training techniques are often used to improve life quality and increase well-being. Given the effect of these techniques on brain activity, as well as on attention and self-regulation, their use to reduce ADHD symptoms and improve life quality in these patients is currently under study [172]. 

Mindfulness can be defined as paying attention to the present, an activity that implies sustained attention. A systematic review on the effects of mindfulness-based interventions on ADHD found that such approaches were popular among adults with ADHD, finding improvements in attention, although the effects of such approaches in children and adolescents are still unclear [173]. A recent meta-review reported a large effect size of mindfulness on ADHD [174]. A review on the effects of mindfulness-based cognitive therapy on ADHD adults reported good effects of this therapeutic approach, especially when used in conjunction with pharmacological therapy [175], while a systematic review analyzing the effects of meditation-based techniques (either on parents and children or on children only) on ADHD children could not draw a clear conclusion regarding beneficial effects [176].

Adult ADHD patients that underwent an 8-week mindfulness awareness practice period presented decreased ADHD, depression, and anxiety symptoms [177]. Similarly, a study performed with children revealed that an 8-week period of mindfulness-oriented meditation produced improvements in the performance of neuropsychological tests, as well as in ADHD symptoms. Although encouraging, the authors stated that the results are still preliminary, given the small number of children participating in the study [178]. There are also results indicating that this technique produces an improvement in ADHD symptoms in ADHD children with oppositional defiant disorder [179].

#### 6.2.4. Neurofeedback

Neurofeedback (NFB) is a therapy in which patients learn to modify EEG patterns through operant conditioning. There are articles reporting the induction of plastic changes after NFB training [180,181,182,183], supporting a theory explaining the effects of NFB on different brain disorders through the induction of synaptic plasticity, leading to an homeostatic set point. Additionally, besides some unusual cases of headache, no collateral effects have been reported with this technique. One of the most interesting aspects of NFB is the induction of plastic changes from within the brain under normal physiological conditions, without the need for an external stimuli such as pharmacological treatments or transcranial stimulation to alter brain activity, thus the probability of adverse effects is minimal [181].

Specific NFB protocols have been developed over the decades. These protocols were designed based on articles reporting specific qEEG variations in neurological patients or qEEG patterns associated with cognitive function. Some of these standardized protocols have been studied in terms of their ability to treat ADHD [184].

Several articles have addressed the use of NFB in ADHD patients. The results have been mixed and numerous meta-analyses have been published on the subject. The conclusions of these meta-analyses have also been mixed. There are meta-analyses reporting good effects of NFB on ADHD [185,186,187], not finding reliable effects [188], not reaching a conclusion on the subject of efficacy [189], finding a minor effect of this therapeutic approach significantly below what is observed with pharmacological treatment [190], or finding a minor effect only in the presence of pharmacological treatment [191].

An overview of recent publications gave the same impression. Some reports found effects of NFB theta/beta or theta/alpha protocols on ADHD, measurable at follow-up 8 weeks or 12 months after treatment completion [192,193]. Other reports found no effect [194,195,196]. Moreover, there are reports revealing a minor effect of NFB, below the effect levels of other therapeutic approaches [197,198,199].

NFB is a therapeutic approach widely studied for ADHD treatment. The results so far have been mixed. However, given the absence of side effects and its ability to induce synaptic plasticity [181], it is an option worth keeping in mind. 

#### 6.2.5. Other Non-Pharmacological Approaches

The use of non-pharmacological supplementations, such as polyunsaturated fatty acids, peptides, amino acids, plat extracts, probiotics, micronutrients, and herbal supplementation, is currently being studied in order to determine their usefulness in treating ADHD. However, further research is still needed in this area [200].

A study performed in ADHD children under methylphenidate treatment for whom zinc supplementation was added reported no significant effect of zinc supplementation on the total score for a parent’s questionnaire for ADHD or in the hyperactivity and impulsivity subscales. However, zinc-supplemented children present improvements in inattention scores [201].

A meta-analysis on non-pharmacological interventions for ADHD patients found that physical exercise produced a good effect on ADHD cognitive symptoms, especially aerobic exercise targeting executive functions [169].

## 7. Treatment Personalization

In the first sections of this review, we addressed some of the factors associated with ADHD incidence, ranging from a variety of environmental factors to the presence of different genetic polymorphisms. However, these different etiologies are not always present, since patients might present one or another (see Section 2 and Section 3). Similarly, while there are some changes in brain activity associated with ADHD, they are not always the same (see Section 4). Accordingly, there is also variation in the response of patients to both pharmacological and non-pharmacological treatments (see Section 5).

Since the etiology of ADHD could be very different from patient to patient, the precise nature of the physiological changes underlying the clinical manifestations of ADHD in each case could be slightly different, affecting the effectiveness of the chosen treatment and possibly explaining the variation in the effect of the same treatment on different patients. This can be observed in the variations in qEEG activity observed in different studies [112,113,114,116,121]. However, the design of personalized treatments based on specific characteristics of each patient could lead to better clinical results. In this regard, strategies to adjust therapeutic approaches based on patients’ characteristics have been used for both pharmacological and non-pharmacological therapies.

Selecting the appropriate pharmacological treatment and the dose to be used takes some time, given the large inter-individual variability regarding treatment efficacy, leading to a delay in reaching a therapeutic effect, and in some cases producing an early termination of treatment due to frustration, either by the provider or the family [157]. Moreover, there is some variability regarding patient response to methylphenidate, including patients that do not achieve adequate symptom control or experience adverse effects with commonly used doses. Therefore, dose optimization has been proposed as a means to achieve an adequate effect for most of the patients, enhancing both the efficacy and safety of methylphenidate treatment [3]. This has led to the search for strategies to find adequate treatments for each patient, such as pharmacogenomics, in which a patient’s genotype for a particular gene is used to predict the effects of medication in that patient. However, in spite of the progress that has already been made, no pharmacogenomic test so far has been found to be helpful in treatment selection [157].

Treatment resistance has been reported for both atomoxetine [158] and methylphenidate [121,159,160]. For this reason, qEEG can be used as a source of information to determine at an earlier point whether methylphenidate [121,160,202] or atomoxetine [107,202] is effective or if an alternative treatment is needed for a patient.

Most of the reports on the use of NFB for ADHD use a standardized protocol, either equal for all participants or adapted to each patients after qEEG analysis. However, there is another more personalized approach known as qEEG-informed (or qEEG-guided) NFB. In this variant of NFB, rather than selecting a particular protocol (for example, theta/beta ratio) and applying it to all participants, subjects receive a NFB protocol selected for them after qEEG analysis. This type of NFB has been successfully used in schizophrenia [203], obsessive compulsive disorder [204], migraine [205], dementia [206], and with learning-disabled children [207,208].

There are so far only two studies applying qEEG-informed NFB in ADHD patients, so it is not yet possible to perform a meta-analysis on the effects of this type of NFB on ADHD. However, a positive effect of NFB has been reported in both published studies [209,210]. 

## 8. Discussion

ADHD is an NDD with a complex etiology. While it is clear that its main cause is alterations in neurodevelopmental processes such as synaptogenesis, myelination, and neurogenesis [5], the causes of these neurodevelopmental alterations are diverse. In some cases they might be associated with environmental factors such as premature birth [6], perinatal problems [9], nutrition during pregnancy [10], or exposure to heavy metals [17,18,19,26,27,29]. Additionally, there is strong evidence of genetic influence on ADHD [43,44], and an interaction between environmental and genetic factors cannot be discarded.

The purpose of this review is not to fully describe all factors associated with ADHD appearance, but rather to address some of the main etiologies described so far, in order to clarify the high diversity of factors associated with this NDD. When analyzing the different sections of this review, one thing becomes evident—that ADHD patients are diverse regarding the etiology of their condition and their responses to treatment. This heterogeneity outlines the high variability in patients’ particular conditions regarding ADHD symptom manifestation and treatment, since it is probable that the underlying neurophysiological alterations for each patient are at least slightly different. Thus, standardized treatment (either pharmacological or non-pharmacological) may not be equally efficient in all cases. 

Moreover, there could be other factors that are usually disregarded in relation with ADHD incidence, but which might play an important role in this condition. Recently, the gut microbiome has been the subject of intense research as an ADHD-associated factor, and even though further research is needed in order to determine its precise influence on ADHD, there are already reports indicating a possible link between them [211,212,213,214].

In the end, all of these factors produce changes in brain structure and function [1,7,112,113,114,115], leading to the symptomatology observed in ADHD patients. Therapeutic approaches to treat this condition have the objective of compensating such alterations in order to reduce symptoms and improve quality of life. However, as we have observed in this review, not all patients present the same neurophysiological changes. Studies performed on qEEG activity have yielded different results regarding brain electrical activity in ADHD patients [4,112,114,120]. Additionally, both brain imaging and qEEG techniques have revealed that changes are not consistent throughout the lifespan, being different in children and adults [114,115,116,121]. Therefore, there is a need for treatment personalization for each ADHD patient in order to achieve greater effect with minimal adverse effects.

Pharmacological treatments, both stimulants and non-stimulants have proven to be effective and safe for ADHD patients [132,133], and thus are widely prescribed to treat this condition. However, the pharmacological approach to ADHD treatment has some drawbacks, mostly regarding difficulties in reaching effectiveness in all patients [3,121,157,158,159,160] and the presence of adverse effects [133,135,144].

The search for other therapeutic options has led to the assessment of the effects of other drugs on ADHD [161], as well as the design of non-pharmacological treatments, such as behavioral parent training, CBT, attention-improving techniques, and NFB. 

The effects of behavioral parent training on ADHD symptoms in children are not consistent, with some articles finding effects [163] and others not finding any [162]. However, behavioral parent training does reduce stress in parents and promotes a better coexistence at home, which is favorable for children [162,165]. In the case of CBT, there is more evidence indicating a good effect in reducing ADHD symptoms [167,168,169,170,171]

Attention training techniques are still under intense study. There is some evidence regarding the effect of this technique on ADHD in adults [173,175], while in children and adolescents the results are not clear so far [173,176].

A number of studies on the effect of NFB on ADHD symptoms have yielded different results, either finding a positive effect [185,186,187,192,193], a mild effect [190,197,198,199], or no effect at all [188,194,195,196]. However, NFB has a number of advantages that encourage the search for an adequate protocol to treat ADHD patients. It is targeted directly to change brain activity associated with the condition under treatment, it has virtually no side effects, and the therapeutic effect is due to the induction of plastic changes in the central nervous system, thus it might establish a long-term changes [180,181,182,183]. 

## 9. Conclusions

In the present review, we have gone through some of the factors associated with ADHD, and it is clear that a great heterogeneity exists in the etiology of this condition. Therapeutic approaches, although functional in many cases, also show heterogeneity in their effects in certain groups of patients. The diverse range of effects of the therapeutic approaches used should not be a surprise, given the diversity of etiologies found in ADHD. Even though clinical manifestations of this condition might be similar (diagnosis is based on the presence certain symptoms), the same clinical manifestations could occur with different underlying physiological changes, considering the variations in qEEG activity in different groups of patients [112,113,114,116,121]. Thus, these neurophysiological changes presented by patients may not necessarily respond in equal form to a given therapeutic approach. Given the inter-personal variance in the etiology of ADHD, it is advisable to personalize the therapeutic approach. Regarding pharmacological therapies, dosage optimization [3], pharmacogenomics [157], and the use of qEEG to select the adequate drug for a given patient have been proposed [107,121,160,202].

Regarding non-pharmacological options, the use of qEEG-informed NFB has been proposed for personalized treatment in ADHD patients. The studies carried out to date have shown positive results [209,210], although the number of studies is still too small to draw a conclusion. However, given the advantages of NFB [181] and the positive effects of this approach reported for other conditions [203,204,205,206,207,208], it is worth performing further studies on the effectiveness of this type of NFB on ADHD.

## Figures and Tables

**Table 1:** Polymorphisms of the BDNF gene studied in relation with ADHD incidence. * Polymorphisms for which contradictory results have been reported. rs, reference SNP ID number.

Polymorphism	Positive Association with ADHD	No Positive Association with ADHD
Val66Met (rs6265) *	[57,58,59]	[46,60,61,62,63]
C270T (rs27656701)	[58,61]	
rs11030101	[62,64,65]	
rs10835210	[62,63]	
rs12291186		[63]
rs7103411		[63]
rs7103873		[62,63]
rs2030324 *	[57,58,59,64]	[46,60,61,62,63]
